# Oral Squamous Cell Carcinoma in a Middle-Aged Patient: Case Report and Literature Review

**DOI:** 10.7759/cureus.96192

**Published:** 2025-11-06

**Authors:** Yuthika Yadav, Sanjana Malhotra, Girik Subudhi, Prachee Jha

**Affiliations:** 1 Medicine and Surgery, Subharti Medical College and Chhatrapati Shivaji Subharti (CSS) Hospital, Meerut, IND; 2 Oral Medicine and Radiology, Subharti Dental College and Hospital, Meerut, IND; 3 Medicine and Surgery, Rohilkhand Medical College and Hospital, Bareilly, Bareilly, IND; 4 Oral and Maxillofacial Surgery, Dr. Prachee’s Radiance Dental, Cosmetology and Implantology Centre, Navi Mumbai, IND

**Keywords:** carcinoma, oral cancer, potentially malignant disease, squamous cell, tobacco

## Abstract

Knowledge of the epidemiology of oral malignancies, especially of squamous cell carcinoma in the oral cavity, allows for lesser morbidity and mortality and the formulation of appropriate, prompt, treatment programs. These regional differences are of clinical interest as they often shape both diagnostic approach and therapeutic decision-making. Surgery relics the main procedure for oral cavity carcinoma, and reconstruction is necessary to repair the tremendous deficiencies that develop after the removal of the tumors themselves. Despite considerable advances in diagnostic and therapeutic techniques, oral cancer continues to present significant clinical and public health challenges. Initial recognition of oral cancer possibly malignant lesions has prevented effective treatment. This case focuses on the medical features of squamous cell carcinoma in the oral cavity, with strategies for primary detection in addition to prevention of this disease, and describes an early-stage squamous cell carcinoma in the oral cavity.

## Introduction

Oral squamous cell carcinoma (OSCC), the furthermost prevalent oral melanoma worldwide, is a cancer of the mouth [1]. About 90% to 95% of all malignant tumors of the oral cavity are squamous cell carcinoma (SCC), which occurs most often in the tongue, particularly in its lateral posterior margin. Typically, males over 50 are affected, and the majority have a history of heavy alcohol and tobacco use [2,3]. Head and neck tumors are the most prevalent kind of tumor, and oral cavity carcinomas are the most frequent type, with around 48,000 cases reported annually [4]. Although many therapy approaches are available, the overall survival rate after five years for OSCC is still only about 50%. In fact, with cancer being treated at an earlier stage, the survival rates can be as high as 80% [5]. Although OSCC affects males more than females, its prevalence and mortality rates increase with patient age [6].

Oral Mucosal Conditions, which are at a higher risk of becoming cancer, are called potentially malignant disorders (PMDs) of the oral mucosa [3]. Discoid lupus erythematosus, dyskeratosis congenita, cheilosis, oral submucous fibrosis, erythroplakia, actinic, palatal keratosis in reverse smoking, epidermolysis bullosa, oral lichen planus, and dyskeratosis congenita constitute oral PMDs [7]. Numerous etiologies for the above-mentioned disorders exist, including genetic abnormalities predisposing to transformed tissue regeneration, hereditary diseases, and illnesses secondary to exogenous factors such as HPV, liquor, tobacco, cannabis use, chronic inflammation, as well as immune-mediated conditions [8]. Therefore, the general public and medical personnel should be better educated on the symptoms, signs, and the need for immediate referral and biopsy of suspicious lesions of OSCC. With these initiatives, for those with OSCC, better treatment outcomes, earlier detection, and finally higher survival rates can be achieved [9].

This epidemiological change underscores the evolving risk profile and stresses that individuals who are genetically or environmentally predisposed can develop malignant transformation even after brief exposure to carcinogenic behaviours [7,8]. This particular case is significant because it concerns a middle-aged patient who developed an early-stage OSCC of the buccal mucosa despite having a relatively brief history of tobacco use. Regardless of the length or severity of risky behaviours, this emphasises the vital need for increased clinical vigilance, early screening, and timely diagnosis of oral lesions in all age groups. This case is being reported to emphasise the significance of early detection and multidisciplinary treatment of OSCC, particularly in cases with atypical presentations involving younger or intermittent tobacco users.

## Case presentation

A 35-year-old male underwent evaluation at the Department of Oncosurgery with the foremost complaint of five days of slight pain in addition to swelling in his lower right back tooth. The patient had the habit of chewing tobacco for three years. On inspection, an ulcer - proliferative growth involving the lower right muco-buccal fold, which was yellowish to white. The size of the lesion was about 3 cm, extending from the right 1 molar region up to the right 3 molar region in the muco-buccal fold. On palpation, the growth was firm and tender; slight bleeding was also present. The lesion had fissures and was ulcerated in nature and yellowish-white in color. The patient was otherwise well, and his medical history was not contributing. The patient gave no history of other parafunctional habits. No aggravating or relieving factors were associated. The oral hygiene status of the patient was fair. The patient did not have any history of cough, expectoration, dyspnea, edema, vomiting, allergy, diabetes, hypertension, fainting, convulsions, or diarrhea.

Extra oral examination

**Figure 1:**
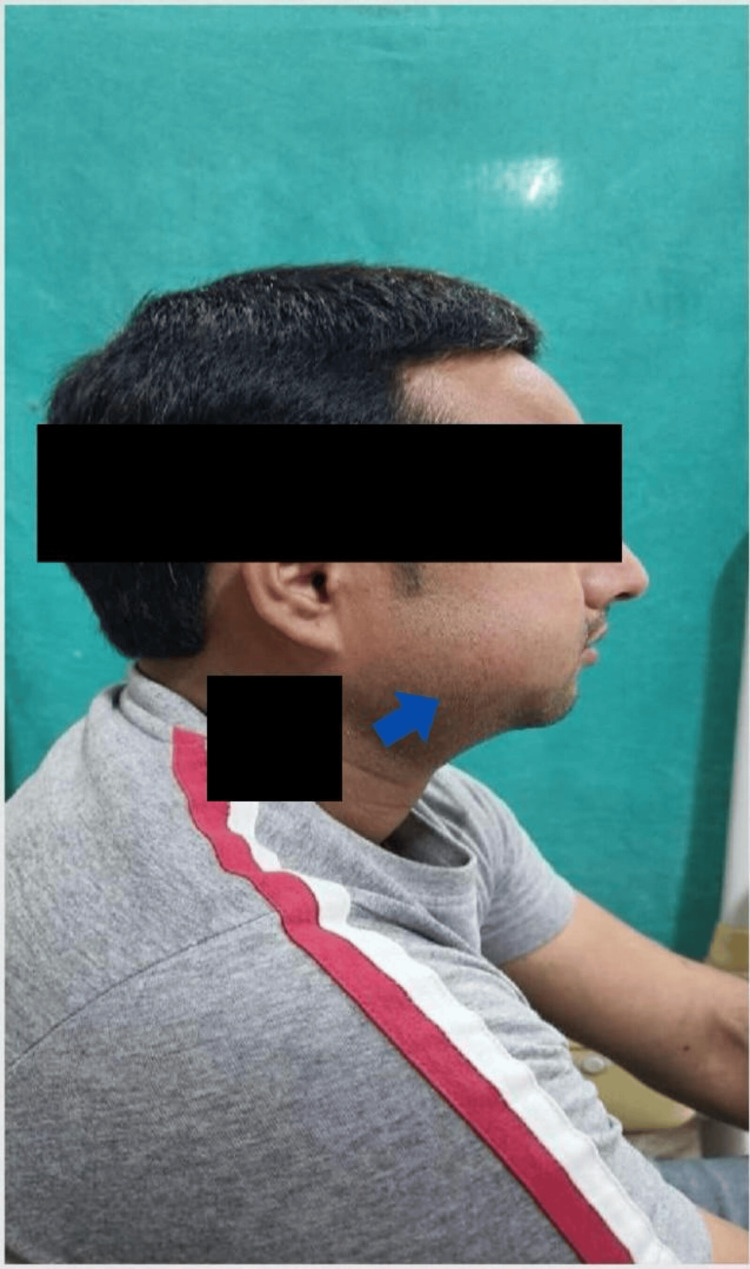
External Oral Examination Clinical photograph showing diffuse swelling on the right side of the face. Palpation revealed firm, tender, and palpable right submandibular lymph nodes indicative of regional lymphadenopathy.

On further oral examination, it was noticed that the patient has an asymmetrical face with reduced mouth opening; in addition, the range of movements was reduced. However, clicking and deviation were absent. Diffuse, firm, and palpable inflammation on the right side of the face was present. The right submandibular lymph nodes were tender and palpable (Figure 1). 

Intraoral examination

**Figure 2:**
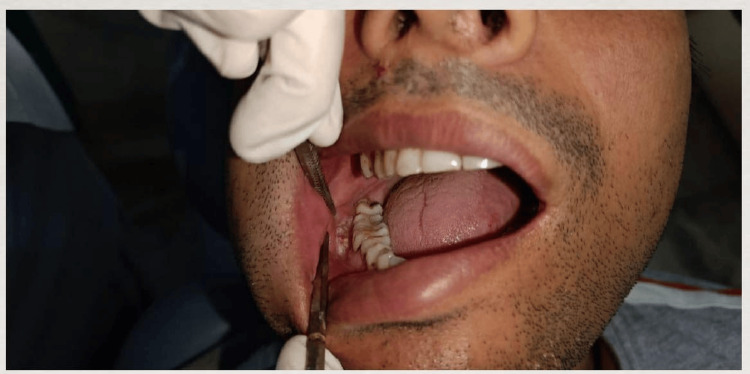
Patient Exhibiting Angle’s Class 1 Molar Relationship

On intra-oral inspection, it was noticed that the right buccal mucosa was soft and edematous. However, the left buccal mucosa, palate, tongue, palate of the mouth, and alveolar mucosa were found to be normal. The periodontal status of the patient was good since bleeding on probing, recession, pockets, trauma from occlusion, crowding, attrition, abrasion, and erosion were absent. The patient exhibited Angle’s Class 1 Molar Relationship [10] (Figure 2).

Details of the patient’s history and preoperative findings are summarized in Table 1.

**Table 1:** Pre-Anaesthetic Evaluation and Clinical Summary Summary of pre-anaesthetic evaluation parameters and clinical history before surgery. It summarizes key preoperative parameters including vital signs, comorbidities, substance use history, and baseline health status to aid in perioperative risk evaluation and management planning.

CATEGORY	PARAMETER	FINDINGS/NOTES
Patient Details	Age/Sex	38 / M
	Weight	65 kg
	Date	23/07/25
	Diagnosis	Carcinoma of Buccal Mucosa
	Operation Proposed	Commando procedure
History	Cough	Present
	Expectoration	Present
	Dyspnea	Absent
	Exercise Tolerance	Good
	Asthma	Absent
	Smoking	Not documented.
	History of tobacco chewing:	Present for approximately 2 years.
	Alcohol	Regular intake for the past 2 years.
	Drug Therapy	NA
	Previous Illness/Surgery	NA
	Pain Chest	Absent
	Palpatations	Absent
	Cyanosis	Absent
	Allergy	NA
	Back Problems	NA
	Diabetes/Hypertension	No History of DM or Hypertension
	Bleeding Disorder	None
	Family H/O Malignant Hyperpyrexia	No significant family history
Chief Complaint		Lump in Right Buccal Mucosa

General and systemic examination

A mesomorphic body build was indicated by the patient’s height of 178 cm and weight of 65 kg, as determined by a general examination. Vital parameters were within normal limits, with a pulse rate of 76 beats per minute, blood pressure of 110/70 mmHg, body temperature of 98.6°F, and respiratory rate of 18 breaths per minute. There was no evidence of jaundice on examination of the nails and sclera, and no clinical signs of clubbing or cyanosis were observed. Systemic examination revealed bilateral air entry to be normal (respiratory System); the patient was conscious and oriented (CNS); S1 and S2 were present with no murmurs heard (CVS); and both spine and gait were within normal limits. 

Further detailed findings from the preoperative general and systemic examination are summarized in Table 2. 

**Table 2:** General and Systemic Examinations This table summarizes the findings of the preoperative general and systemic examination. Reference ranges are provided for measurable parameters to aid in clinical interpretation.

CATEGORY	PARAMETER	FINDINGS / NOTES	REFERENCE RANGE/NORMAL VALUE
General Examination	Pulse	76 bpm	60–100 bpm
	Blood Pressure	134/89 mmHg	90–140 / 60–90 mmHg
	Temperature	Afebrile	97°F–99°F
	Pallor	Absent	Absent
	Clubbing	Absent	Absent
	Cyanosis	Absent	Absent
	Icterus	Absent	Absent
	Jugular Venous Pressure (JVP)	Normal	Normal
	Oral Hygiene	Average	Good / Average
	Loose Teeth	None	–
	Artificial Denture	None	–
	Jaw Movement	Adequate	Adequate
	Neck Movement	Adequate	Adequate
	Mallampati (M.P.) Grade	III	I–IV (Grade I–II: Easy airway)
	Inter-incisor Gap	3.5 cm	>3 cm (Normal)
	Thyromental Distance	6.5 cm	>6 cm (Normal)
	Intubation Difficulty	Anticipated: Easy	–
	Airway	Patent	Patent
	Psychological Status	Calm and cooperative	Calm and cooperative
Systemic Examination	Respiratory System	Bilateral air entry present, clear; no added sounds	Normal air entry; no added sounds
	Central Nervous System (CNS)	Conscious, oriented	Normal
	Cardiovascular System (CVS)	S1, S2 heard; no murmurs	Normal heart sounds; no murmurs
	Spine	Within normal limits	Normal
	Gastrointestinal Tract (GIT)	Within normal limits	Normal

Preoperative investigations were conducted to assess the patient’s general health status and suitability for anaesthesia. Laboratory results, including haemoglobin, renal function tests, and serum electrolytes, were within normal limits. Coagulation profile and cardiovascular evaluations (ECG, chest X-ray, and echocardiography) revealed no abnormalities. The patient was classified as ASA Grade II and deemed fit for general anaesthesia. Pre-anaesthetic instructions included nil per oral status after midnight, arrangement of adequate blood levels, and administration of premedication as advised. The detailed results of the preoperative investigations and anaesthetic assessment are presented in Table 3. 

**Table 3:** Preoperative Investigations and Anaesthetic Assessment This table summarizes the results of preoperative investigations and anaesthetic assessment, highlighting the patient’s stable biochemical profile, absence of systemic abnormalities, and readiness for general anaesthesia. PCV: packed cell volume, LFT: liver function test, ABG: arterial blood gas, BT: bleeding time, CT: clotting time, ECG: electrocardiogram, ASA: American Society of Anesthesiologists, CBC: complete blood count, PT: prothrombin time, INR: international normalized ratio, RBS: random blood sugar, CXR: chest x-ray, OR: operating room, PAC: pre-anesthetic checkup.

CATEGORY	PARAMETER	FINDINGS/NOTES	REFERENCE RANGE/NORMAL VALUE
Investigations	Haemoglobin/PCV	15.4 g/dL	13–17 g/dL (Males)
	Blood Urea	25.1 mg/dL	15–40 mg/dL
	Serum Creatinine	0.87 mg/dL	0.7–1.3 mg/dL (Males)
	Serum Sodium (Na⁺)	145 mEq/L	135–145 mEq/L
	Serum Potassium (K⁺)	3.9 mEq/L	3.5–5.0 mEq/L
	Serum Chloride (Cl⁻)	99 mEq/L	98–107 mEq/L
	Serum Calcium (Ca²⁺)	8.8 mEq/L	8.5–10.5 mEq/L
	LFT/ABG/Thyroid	Not done / Normal	Normal values
	BT/CT	Normal	BT: 1–7 min; CT: 3–10 min
	ECG/X-Ray Chest/Echo	Normal findings	Normal
Pre-Anaesthetic Advice	Nil orally after	12 AM (midnight) before surgery	–
	Blood Required	Arranged	–
	Premedication	As advised	–
	Informed Consent	Obtained	–
Planned Anaesthesia		General Anaesthesia (GA)	–
ASA Grading		ASA Grade II	ASA I–V
Instructions		CBC, PT, INR, RBS, LFT, ECG, CXR to be done	–
		Arrange adequate blood and inform OR on call	–
		Routine PAC advised	–

Examination of the area of interest

Inspection

Ulcero-proliferative growth involving the lower right muco-buccal fold, which was yellowish to white in colour. The size of the lesion was about 3 cm, extending from the 1 molar region up to the 3 molar region in the muco-buccal fold.

Palpation

On palpation, the growth was firm and tender; slight bleeding was also present. The lesion was ulcerated in nature and had fissures.

Exfoliative cytology and toluidine blue staining were performed as a chairside study. These diagnostic tools were chosen for their usefulness and value in clinical settings with available resources. Toluidine blue staining was prioritized as a quick chairside screening method for spotting dysplastic or malignant mucosal lesions. This helped in deciding whether to perform an incisional biopsy. Exfoliative cytology was done to provide a cytological check before confirming with histopathology. We performed computed tomography (CT) scanning to clarify the local extent of invasion and check for nodal involvement, which was important for planning the commando procedure. An H&E-stained smear of the right buccal mucosa's exfoliative cytology revealed sheets of pleomorphic epithelial cells with hyperchromatic nuclei and a change in the nuclear-cytoplasmic ratio. Acute inflammatory cell sheets were also observed.

**Figure 3:**
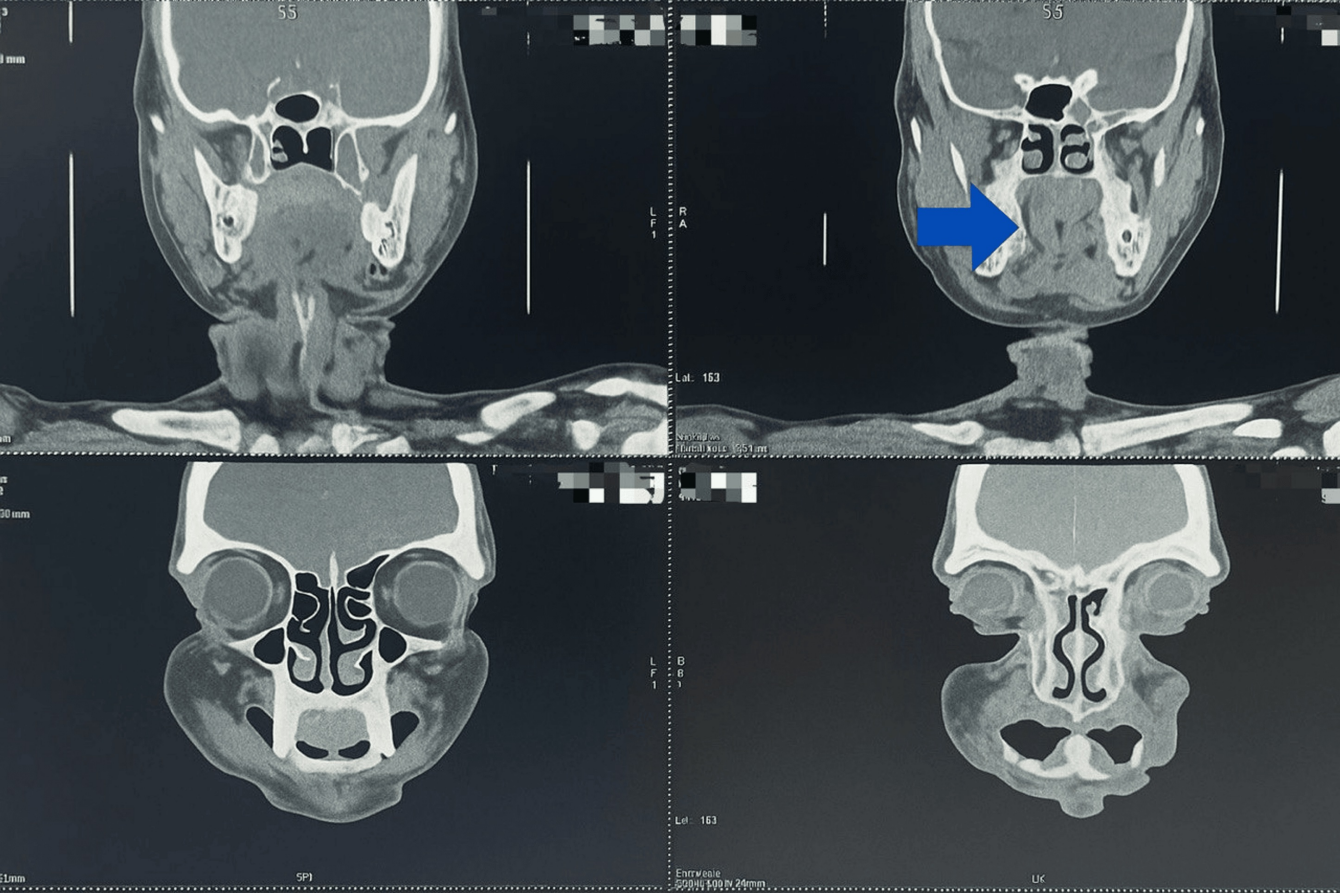
CT Scan Coronal CT scan of the head and neck region showing a soft tissue density (indicated by the blue arrow) extending inferiorly from the skull base. This finding is suggestive of a possible mass lesion, soft tissue swelling, or pathological thickening in the region.

**Figure 4:**
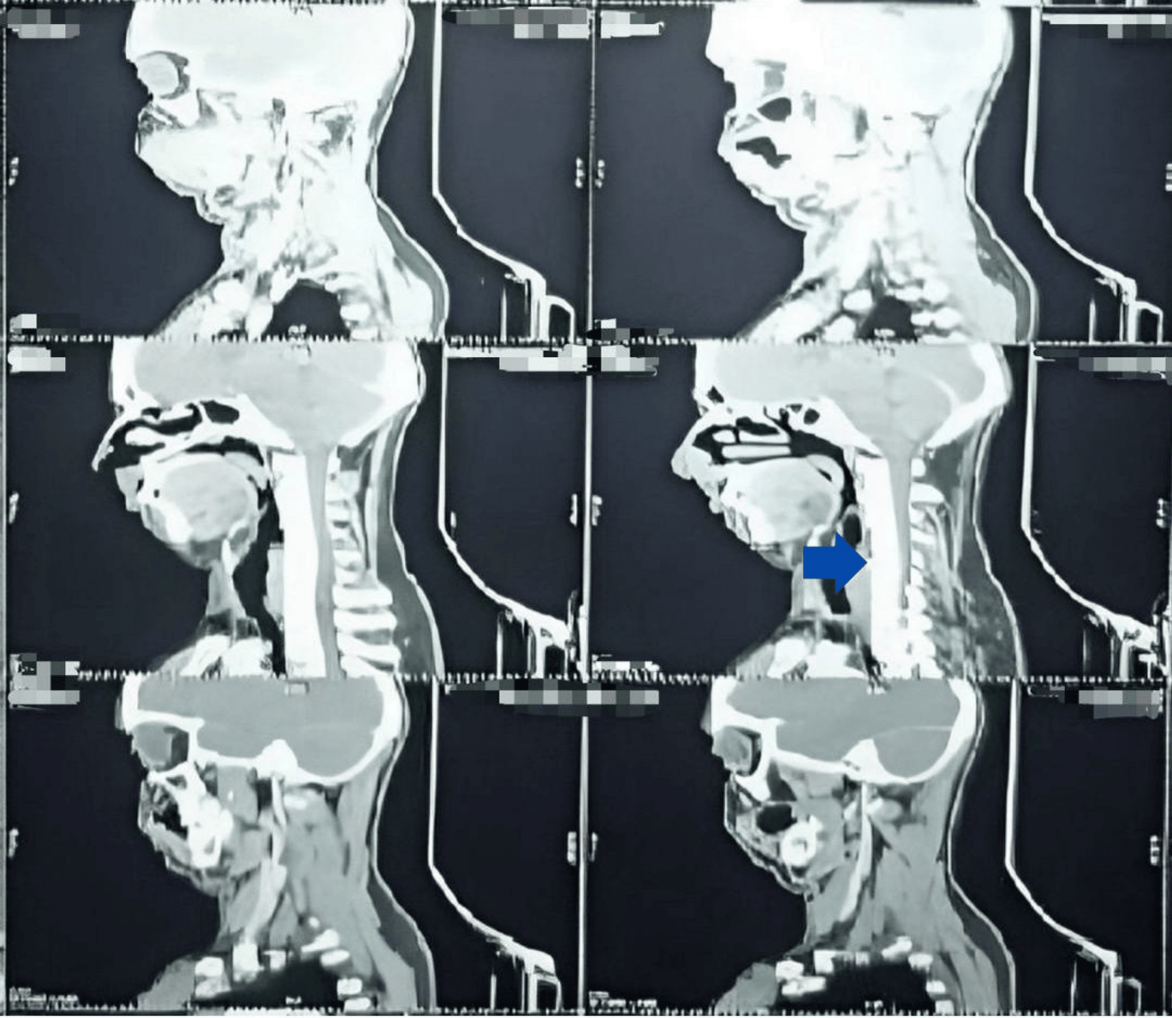
CT Scan Sagittal CT scan shows a soft tissue mass arising from the oral cavity, extending posteriorly into the oropharyngeal and nasopharyngeal regions, causing airway narrowing and infiltration of adjacent soft tissues — features consistent with advanced squamous cell carcinoma (SCC) of the oral cavity with posterior extension.

**Figure 5:**
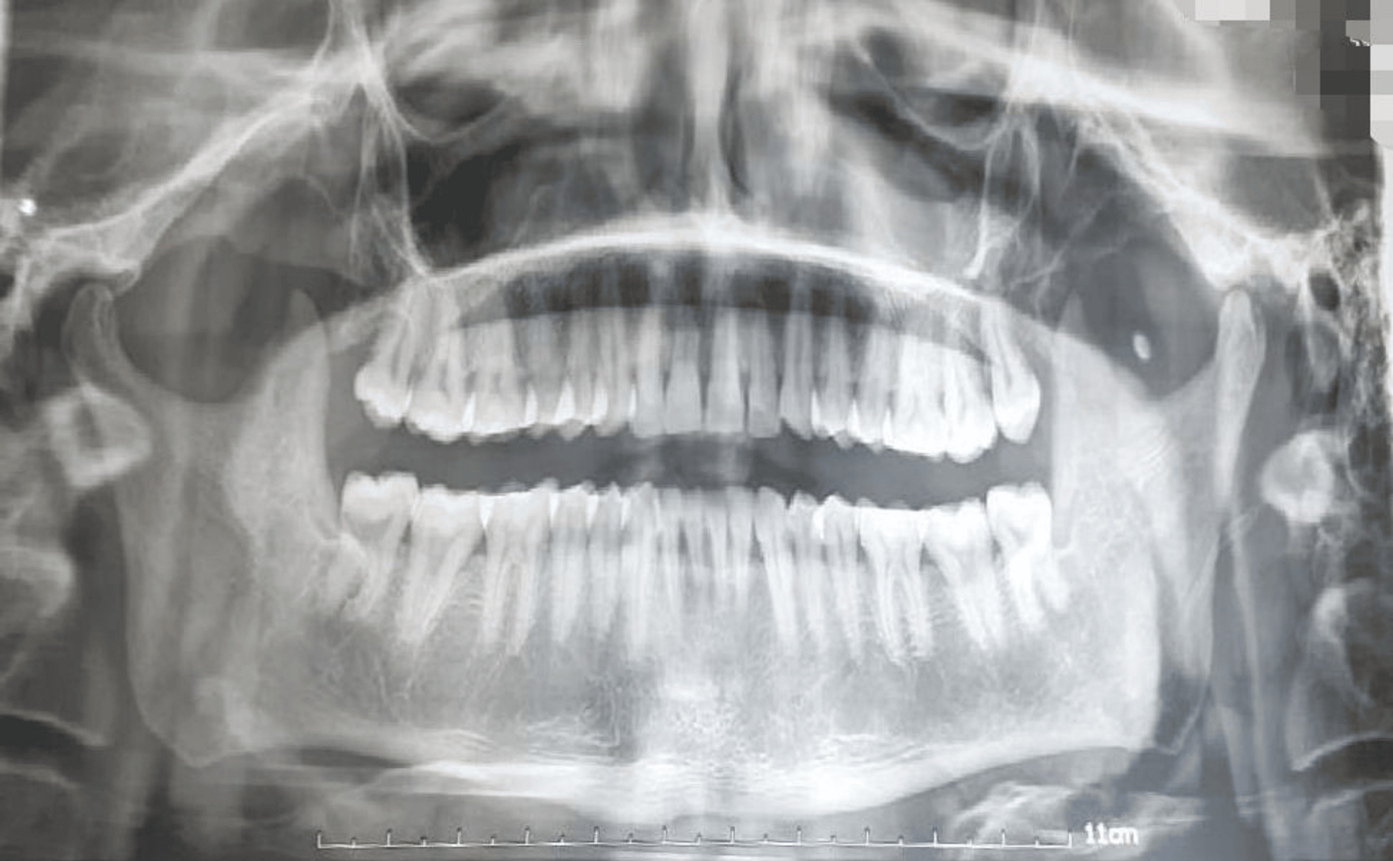
Opthopantogram X-Ray The panoramic radiograph demonstrates all maxillary and mandibular teeth with preserved lamina dura and intact cortical outlines. No obvious osteolytic lesion, cortical erosion, or pathological fracture is visualized. Within the limitations of an OPG, there is no radiographic evidence of bony involvement. (Scale bar=1cm).

CT Scan and orthopantomogram (OPG) of the patient were also done as radiographic examinations. (Figures 3, 4, 5). All radiological and clinical images were verified against original digital imaging and communications in medicine (DICOM) metadata and operative records to confirm correct orientation; no images were inadvertently mirrored.

A provisional diagnosis of malignancy of the right muco-buccal fold was made based on the chief complaint and medical inspection.

**Figure 6:**
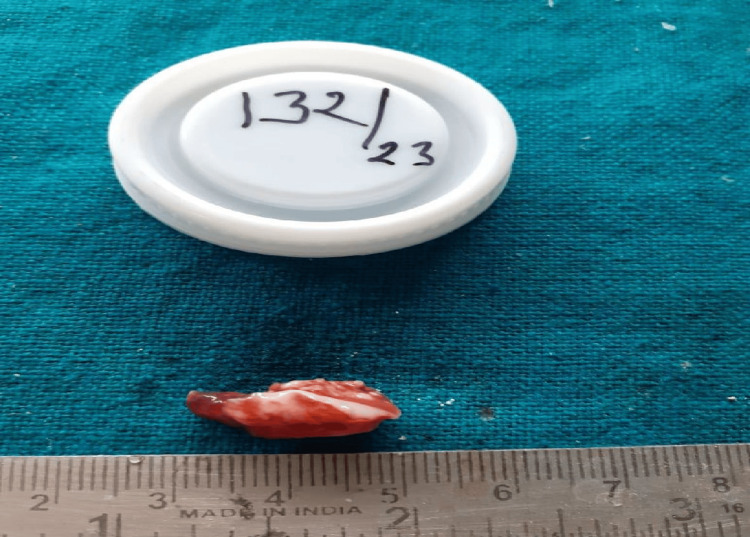
Biopsy Specimen (7×7×7mm) Excised biopsy specimen (7*7*7mm; scale bar=1cm) obtained from the right muco-buccal fold under local anesthesia. The tissue sample was sent for histopathological analysis confirming squamous cell carcinoma.

For a confirmed analysis, the patient was subsequently recommended to have an incisional biopsy and blood tests performed (Figure 6). In blood investigations, every parameter was within normal limits. The right muco-buccal fold was incisionally biopsied during all aseptic procedures under local anesthetic, and the specimen was sent to the Subharti Dental College and Hospital's Oral and Maxillofacial Surgery department in Meerut for histology. 

A soft tissue section stained with H&E revealed dysplastic orthokeratotic stratified squamous epithelium in conjunction with fibrous connective tissue in the right muco-buccal fold. The connective tissue stroma was invaded by invasive sheets and islands of dysplastic epithelial cells that displayed dysplastic characteristics such as nuclear hyperchromatism, aberrant mitoses, cellular and nuclear pleomorphism, altered nuclear-cytoplasmic ratio, a few specific cell keratinizations, and the development of keratin pearls. Blood vessels, extravasated red blood cells, and extensive chronic inflammatory cell infiltrates in the right buccal mucosa were indicative of well-structured squamous cell carcinoma (Table 4).

**Table 4:** Histopathological Features of the Biopsy Specimen from the Right Muco-Buccal Fold Microscopic examination of the H&E-stained soft tissue section revealed dysplastic ortho-keratotic stratified squamous epithelium with invasion into the underlying fibrous connective. tissue stroma. The epithelial islands exhibited nuclear hyperchromatism, pleomorphism, abnormal mitoses, and keratin pearl formation. The presence of chronic inflammatory infiltrate and stromal vascular congestion further supported the diagnosis of well-differentiated squamous cell carcinoma.

HISTOPATHOLOGICAL FEATURE	OBSERVATIONS
Type of epithelium	Dysplastic ortho-keratotic stratified squamous epithelium
Underlying connective tissue	Fibrous connective tissue stroma with inflammatory infiltrate
Pattern of invasion	Invasive sheets and islands of dysplastic epithelial cells
Cytological characteristics	Nuclear hyperchromatism, cellular and nuclear pleomorphism
Mitotic activity	Presence of abnormal mitotic figures
Nuclear-cytoplasmic ratio	Increased
Keratinization	Presence of individual cell keratinization and keratin pearl formation
Inflammatory component	Dense chronic inflammatory cell infiltrate
Final histopathological diagnosis	Well-differentiated squamous cell carcinoma

Treatment

**Figure 7:**
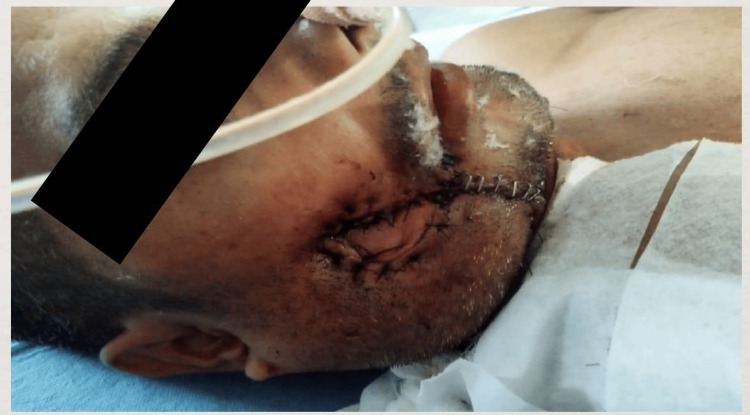
Commando Operation (Right Lateral View)

**Figure 8:**
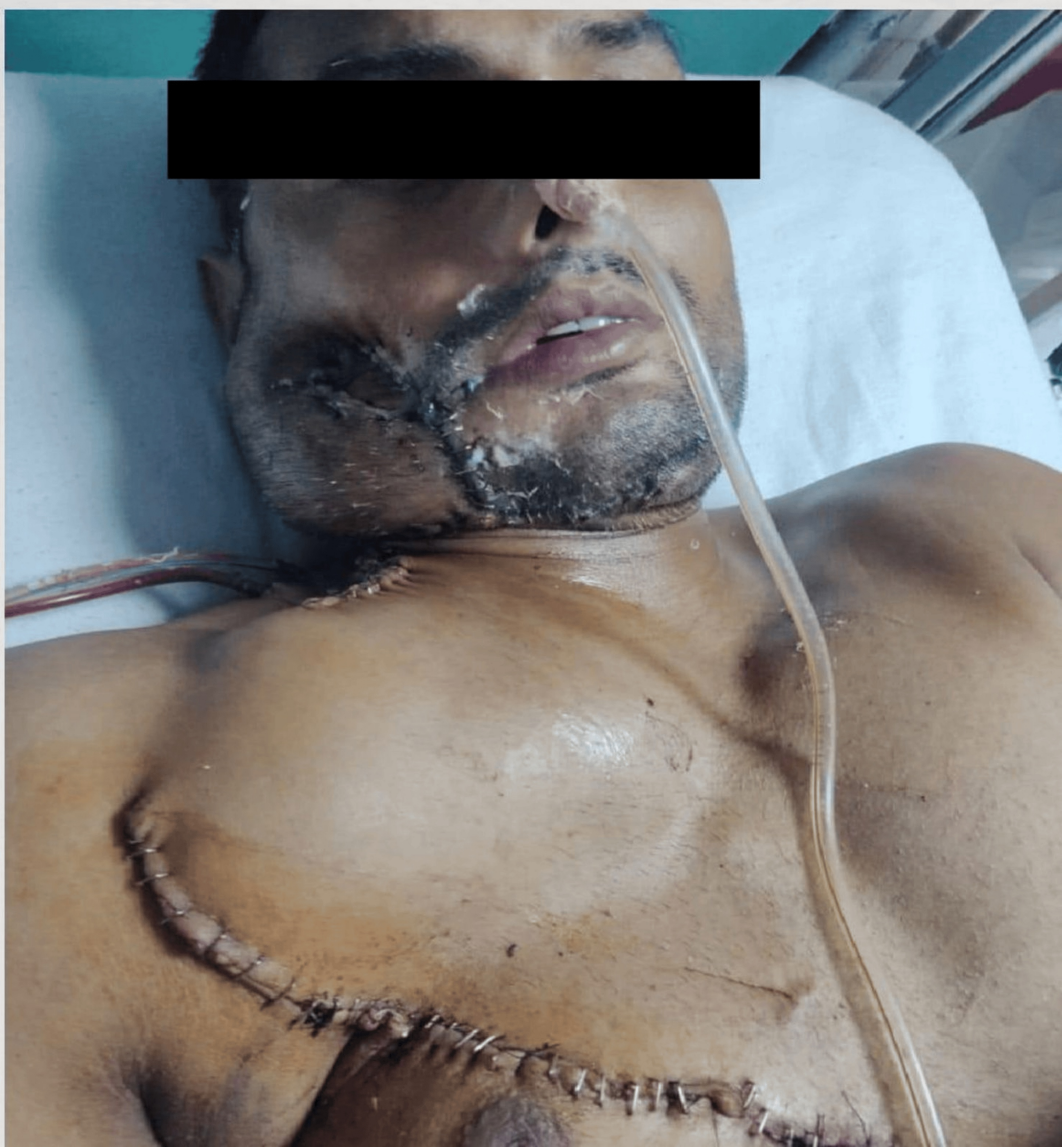
Commando Operation (Right Oblique View) Intraoperative view following commando procedure (right hemimandibulectomy with modified radical neck dissection and pectoralis major myocutaneous flap reconstruction). The postoperative site shows satisfactory flap coverage and hemostasis.

As a treatment modality commando operation was performed under general anesthesia, which included a modified radical neck dissection, right hemi mandibulectomy, and pectoralis major myocutaneous flap (Figures 7, 8).

The patient was discharged with stable vitals, accepting Rt feed well, passing urine, flatus, and stool normally after 10 days of the surgery.

Medications

The patient was prescribed oral medications, including tablet Augmentin 625 mg thrice daily, tablet Pantop 40 mg once daily, Tablet Diclofenac twice daily, and tablet Chymoral Forte thrice daily. Additionally, T-Bact ointment was advised for local application over the suture site. All medications were administered through the Ryle’s tube (RT). Supportive measures included Hinex protein powder mixed with one glass of water thrice daily to ensure adequate nutritional intake. The patient was instructed to maintain proper oral hygiene, perform Betadine gargles thrice daily, and continue with chest physiotherapy and incentive spirometry to aid postoperative recovery and prevent pulmonary complications.

**Figure 9:**
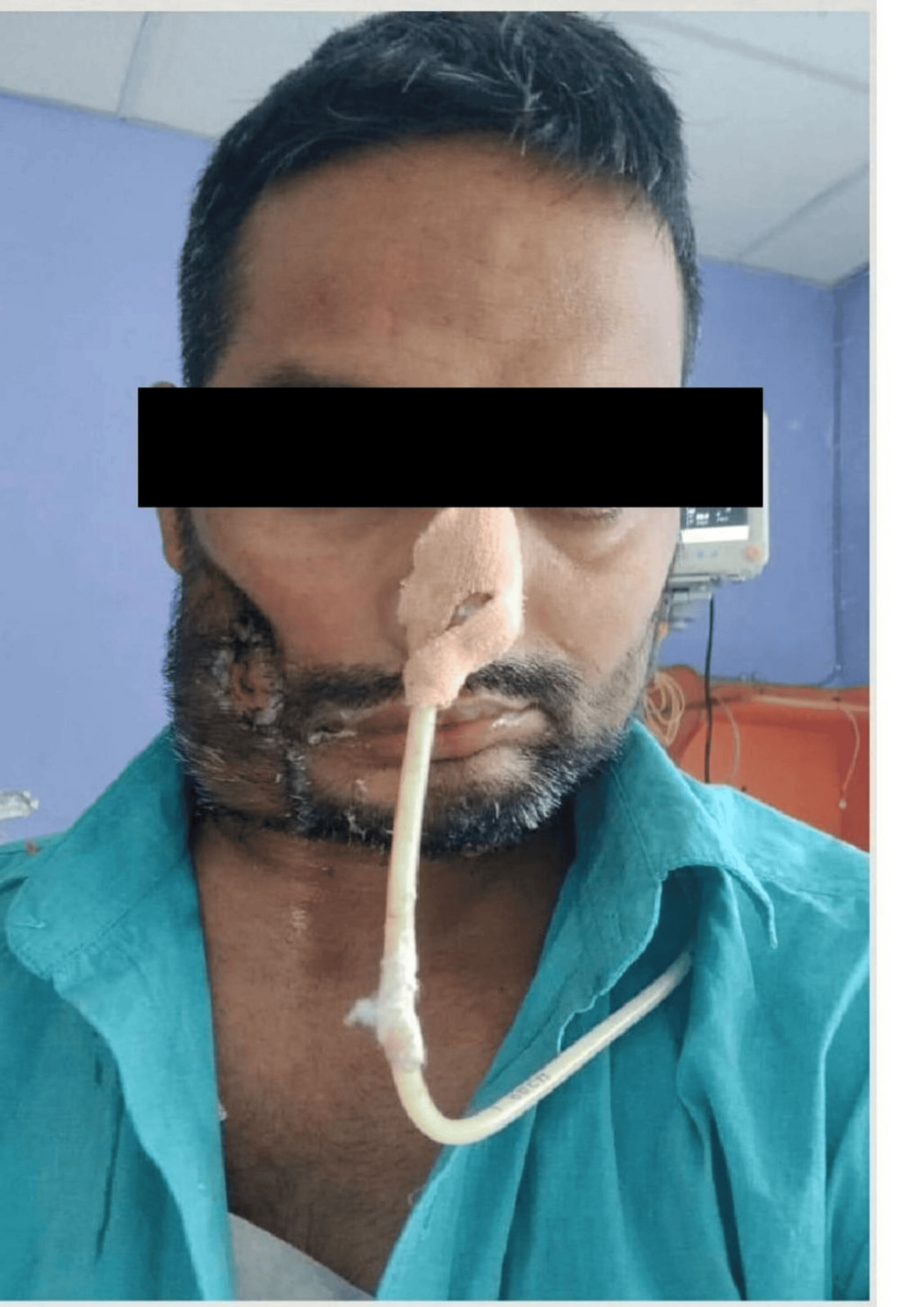
Follow-up of the Patient After a Week Postoperative photograph taken one week after surgery showing stable wound healing, absence of infection, and satisfactory recovery of the reconstructed area.

The first follow-up of the patient was taken after a week (Figure 9).

Oral function gradually improved after surgery, according to the evaluation. After a week, the mouth opening increased from 3.8 cm to 4.5 cm. Clear and understandable speech was used, and aspiration-free swallowing was sufficient. Within three weeks following surgery, the patient was able to return to a semi-solid and then regular diet.

At the three-month follow-up, the patient demonstrated satisfactory healing with stable flap integrity, no evidence of infection or dehiscence, and complete epithelialization of the intraoral site. There was no clinical or radiographic sign of local recurrence. The patient reported satisfactory chewing, speech, and swallowing function, indicating favourable postoperative recovery and rehabilitation.

Statement on Diagnostic Tools and Licensing

No copyrighted or licensed scales, questionnaires, or scoring systems were used in this study. All diagnostic methods (exfoliative cytology, Toluidine blue staining, histopathology, and radiological imaging) are standard, widely available clinical procedures and free to use.

## Discussion

Oral squamous cell carcinoma is the most prevalent (for about 95% of all occurrences of oral cancer) [11,12]. Research by Talabani et al. found that women have a lower prevalence than men and that people over 60 were the most afflicted. In the present case study, the group of young people in middle age was affected by the increase in tobacco smoking at an early age, and the topmost of the overall malignancies was found in the sixth decade of life. Oral cancer has a diagnosis average age of 60, which is usually caused by harmful practices as people get older [13]. The two leading risk factors for OSCC are alcohol use and smoking. There are additional causative factors such as dietary practices, viral infection, and genetic predisposition. Cases with head and neck malignancies that have an early diagnosis without metastases have a 76% survival rate, a 41% survival rate if there are cervical lymph node metastases, and a 9% survival rate if there are metastases behind the neck [14]. If dysplastic oral mucosal lesions are not detected and treated in time, they may progress to OSCCs. The survival period for persons with OSCCs is extended to five decades in stages I and II, associated with stages III and IV. The average survival time for those in the third and fourth stages is six months, and the maximum survival time is one year [15], according to reports. The classic appearance of OSCC in the buccal mucosa is a chronic, excruciating ulcer, combined with induration and infiltration of deeper structures in the oral cavity. They are superficial on some occasions and seem to be spreading out instead of penetrating the depths of the tissues [16]. The ulcerative type of SCC is highly damaging, and therefore, an early and precise diagnosis of OSCC is necessary to improve patient prognosis and survival rates. Treatment of OSCC includes chemotherapy, radiation therapy, surgery, or combinations thereof. Differential diagnoses other than OSCC for the case included verrucous carcinoma, basal cell carcinoma, necrotizing sialometaplasia, primary syphilitic lesion, and pyogenic granuloma. Kroll et al. performed research on 105 individuals with intraoral defects and found a fistula incidence of 24.8%. Mahammad Tahir et al. revealed research based on a review of 10 patients in which 10% of patients who underwent pectoralis major myocutaneous (PMMC) flap, including head and neck reconstruction, were infected [17]. PMMC flaps are often used in head and neck reconstruction because of their proven dependability and versatility [18]. Saliva is a useful bodily fluid for disease diagnostics because it is non-invasive. It is increasingly used in the search for biomarkers for oral cancer [19]. Before practical application, aberrant values need to be identified, and compounds in the saliva of cases with head and neck cancer [20] need to be identified. About half of the cancer cases are detected in the last stages (stage III or stage IV), either due to the unavailability of medical care or because people are unaware of early diagnosis. This article confirms the general data that oral cancer is becoming more common all around the world, including India. Both patient and professional diagnostic delays are the reason that initial detection, as well as prevention of oral cancers, is a major concern. 

Prevention and diagnosis

One strategy for preventing oral cancer is to interview people to find out how they feel about being addicted to different bad habits and how much they know about how behaviors contribute to the occurrence of oral cancer. In light of this, initiatives should be undertaken to raise public consciousness of the adverse consequences of bad habits and to periodically perform de-addiction programs tailored to the needs of the rural populace. Screening people for initial cancer and detecting signs of precancer or potentially malignant disease is another practical and affordable way to lower the overall prevalence of cancer.

## Conclusions

Through prompt diagnosis, patient education, and the encouragement of healthy lifestyle choices, the oral physician significantly contributes to the overall reduction of the burden of oral squamous cell carcinoma. Patient outcomes can be greatly enhanced by preventing the progression of suspicious lesions and possibly malignant illnesses to advanced stages. Comprehensive oral exams, education on the negative consequences of alcohol and tobacco use, and patient encouragement for routine dental checkups should all be prioritized. This case highlights the emergence of oral squamous cell carcinoma in a younger adult with limited exposure to conventional carcinogens, reflecting a changing pattern in disease onset. It reinforces the importance of maintaining clinical vigilance even in patients with short or intermittent tobacco use and demonstrates that early recognition and timely intervention can lead to favourable outcomes.

Even while oral cancers might not be totally preventable, community-based preventative measures, early intervention, and focused screening programs can significantly lower their incidence and severity. Improving patient-centered education and interdisciplinary cooperation between dental and medical practitioners are still essential for reducing disease-related morbidity and raising the standard of living for affected individuals. Integrating routine oral cancer screening into primary healthcare, promoting awareness among young adults, and fostering collaboration between dental and medical practitioners are essential steps toward improving early detection and long-term prognosis.

## References

[1] Adeola HA, Bello IO, Aruleba RT (2022). The practicality of the use of liquid biopsy in early diagnosis and treatment monitoring of oral cancer in resource-limited settings. Cancers (Basel).

[2] Aquino IG, Bastos DC, Cuadra-Zelaya FJ, Teixeira IF, Salo T, Coletta RD, Graner E (2020). Anticancer properties of the fatty acid synthase inhibitor TVB-3166 on oral squamous cell carcinoma cell lines. Arch Oral Biol.

[3] Asio J, Kamulegeya A, Banura C (2018). Survival and associated factors among patients with oral squamous cell carcinoma (OSCC) in Mulago hospital, Kampala, Uganda. Cancers Head Neck.

[4] Balsaraf S, Bhambal A, Chole R (2019). Study of oral potentially malignant disorders related to various risk factors amongst the patients attending hospitals in Bhopal, India. Med Pharm Rep.

[5] Bouquot JE, Weiland LH, Kurland LT (1989). Metastases to and from the upper aerodigestive tract in the population of Rochester, Minnesota, 1935-1984. Head Neck.

[6] Dowling P, Wormald R, Meleady P, Henry M, Curran A, Clynes M (2008). Analysis of the saliva proteome from patients with head and neck squamous cell carcinoma reveals differences in abundance levels of proteins associated with tumour progression and metastasis. J Proteomics.

[7] Mortazavi H, Baharvand M, Mehdipour M (2014). Oral potentially malignant disorders: an overview of more than 20 entities. J Dent Res Dent Clin Dent Prospects.

[8] Fang QG, Shi S, Li ZN, Zhang X, Liua FY, Xu ZF, Sun CF (2013). Squamous cell carcinoma of the buccal mucosa: analysis of clinical presentation, outcome and prognostic factors. Mol Clin Oncol.

[9] Franzmann EJ, Reategui EP, Carraway KL, Hamilton KL, Weed DT, Goodwin WJ (2005). Salivary soluble CD44: a potential molecular marker for head and neck cancer. Cancer Epidemiol Biomarkers Prev.

[10] Hershfeld JJ (1979). Edward H. Angle and the malocclusion of the teeth. Bull Hist Dent.

[11] Friedlander PL, Schantz SP, Shaha AR (1998). Squamous cell carcinoma of the tongue in young patients: a matched-pair analysis. Head Neck.

[12] Hirota SK, Migliari DA, Sugaya NN (2006). Oral squamous cell carcinoma in a young patient: case report and literature review (Article in Portugese). An Bras Dermatol.

[13] LE AK, DE MA LL, BE M, KU MA, CE CR, GA GAÃO L, PI FR (2014). Pectoralis major myocutaneous flap for head and neck reconstruction: risk factors for fistula formation. Acta Otorhinolaryngol Ital.

[14] Llewellyn CD, Johnson NW, Warnakulasuriya KAAS (2001). Risk factors for squamous cell carcinoma of the oral cavity in young people - a comprehensive literature review. Oral Oncol.

[15] Siegel RL, Miller KD, Fuchs HE, Jemal A (2022). Cancer statistics, 2022. CA Cancer J Clin.

[16] Sciubba JJ (2001). Oral cancer. The importance of early diagnosis and treatment. Am J Clin Dermatol.

[17] Tahir M, Ullah T, Khan AT, R R (2005). Clinical evaluation of pectoralis major myocutaneous flap in head and neck reconstruction. J Postgrad Med Inst.

[18] Warnakulasuriya S, Johnson NW, van der Waal I (2007). Nomenclature and classification of potentially malignant disorders of the oral mucosa. J Oral Pathol Med.

[19] Vats R, Yadav P, Bano A, Wadhwa S, Bhardwaj R (2024). Salivary biomarkers in non-invasive oral cancer diagnostics: a comprehensive review. J Appl Oral Sci.

[20] Zargaran M, Eshghyar N, Vaziri PB, Mortazavi H (2011). Immunohistochemical evaluation of type IV collagen and laminin-332 γ2 chain expression in well-differentiated oral squamous cell carcinoma and oral verrucous carcinoma: a new recommended cut-off. J Oral Pathol Med.

